# Impact of age and timing of hip orthosis on treatment outcomes in infants with developmental dysplasia of the hip: A systematic review and meta-analysis^☆^

**DOI:** 10.1016/j.jcot.2025.102944

**Published:** 2025-02-19

**Authors:** Daniela Alessia Marletta, Biagio Zampogna, Gabriele Giuca, Matteo Nanni, Sanzarello Ilaria, Danilo Leonetti

**Affiliations:** a Section of Orthopaedics and Traumatology, Department of Biomedical Sciences and Morphological and Functional Images, University of Messina, 98122, Messina, (ME), Italy; b Department of Orthopaedic and Trauma Surgery, Università Campus Bio-Medico Di Roma, Rome, Italy; c Research Unit of Orthopaedic and Trauma Surgery, Fondazione Policlinico Universitario Campus Bio-Medico, Rome, Italy

**Keywords:** Developmental dysplasia of the hip, DDH, Hip orthosis, Pavlik harness, Treatment timing

## Abstract

**Background:**

Developmental Dysplasia of the Hip (DDH) is a prevalent pediatric condition affecting 1–3% of newborns worldwide. Early treatment is crucial to prevent long-term complications such as residual dysplasia, avascular necrosis (AVN), and osteoarthritis. Despite the widespread use of the Pavlik harness, the optimal timing of hip orthosis initiation remains a topic of debate. This systematic review and meta-analysis evaluate the impact of age and timing of hip orthosis application on treatment outcomes in infants with DDH.

**Methods:**

This systematic review was registered on PROSPERO (Registration No. CRD42025638433). A comprehensive literature search was conducted in PubMed, Scopus, and Cochrane Library for studies published between 2000 and 2024. Twenty-two studies meeting inclusion criteria were analyzed, focusing on success rates, healing times, and complications such as AVN and residual dysplasia. Data were pooled for meta-analysis, and statistical analyses were performed using a random-effects model to assess the impact of treatment timing.

**Results:**

Infants treated before 3 months of age achieved a pooled success rate of 88.79 % (SE: 0.57 %), with lower complication rates, including AVN (0.89 %, SE: 0.18 %) and residual dysplasia (1.80 %, SE: 0.25 %). In contrast, treatment initiation between 3 and 6 months had a slightly lower success rate of 87.78 % (SE: 0.34 %), but with higher AVN (9.66 %, SE: 0.30 %) and residual dysplasia (20.27 %, SE: 0.40 %) rates. The Pavlik harness and Tübingen hip flexion splint were most effective in early-treated cases, whereas later treatment initiation or severe presentations resulted in less favorable outcomes.

**Conclusion:**

Early treatment initiation, particularly before 3 months of age, significantly improves treatment success and reduces long-term complications. These findings emphasize the necessity of early screening and timely intervention to optimize outcomes. Future research should focus on refining treatment protocols for delayed presentations and improving management strategies for severe dysplasia.

**Level of evidence:**

Level IV

## 1 Introduction

Developmental Dysplasia of the Hip (DDH) represents a spectrum of abnormalities in the development of the hip joint, ranging from mild acetabular dysplasia to complete dislocation of the femoral head. Early diagnosis and intervention are critical because untreated DDH can result in lifelong complications, including chronic pain, gait disturbances, premature osteoarthritis, and, in severe cases, the need for total hip replacement.^1^ Despite extensive research, several factors, including the ideal timing for intervention, the type of orthosis used, and patient-specific variables such as age at treatment initiation, continue to generate debate within the clinical community.

### 1.1 Epidemiology and screening

DDH is diagnosed in approximately 1–3% of newborns worldwide, with its incidence varying according to factors such as geographic region, race, and family history.^2^ Early detection is paramount in improving outcomes, as hip development is largely dependent on maintaining the femoral head within the acetabulum during infancy. The introduction of routine screening programs, such as the use of physical examinations (Ortolani and Barlow maneuvers) combined with ultrasound in high-risk populations, has improved early diagnosis rates significantly, but challenges remain, particularly in late-presenting cases.^2^

### 1.2 Orthotic treatment and timing

The treatment of DDH varies depending on the severity of the condition and the age at which it is diagnosed. The Pavlik harness is the most commonly employed orthosis for infants younger than six months, largely because it is non-invasive, well-tolerated, and has demonstrated success in stabilizing the femoral head within the acetabulum.^3^ Studies have shown success rates as high as 90 % when treatment is initiated early, typically before three months of age. This success is attributed to the harness's ability to position the hips in flexion and abduction, facilitating the natural remodeling of the acetabulum.^4^

The type and timing of treatment are especially critical in determining long-term outcomes. Numerous studies have reported that treatment initiated before six months of age significantly improves the likelihood of successful femoral head reduction and reduces the risk of complications such as residual dysplasia and avascular necrosis (AVN).^5^ However, the effectiveness of the Pavlik harness diminishes with delayed treatment, particularly in severe cases (Graf type-III or IV), where the risk of failure increases, and more invasive interventions, such as closed or open reduction, are often necessary.^6^

### 1.3 Severity of dysplasia and AVN risk

In addition to the timing of treatment, the severity of dysplasia plays a crucial role in outcomes. For instance, Graf type-II hips generally respond well to treatment with the Pavlik harness or alternative orthoses like the Tübingen hip flexion splint, with success rates exceeding 90 %.^4^ However, Graf type-IV hips, which present with complete dislocation, often exhibit poorer outcomes, even when treated early, and the risk of complications such as AVN is significantly higher.^7^

The use of the Pavlik harness is not without risks, particularly if treatment fails or is improperly managed. Avascular necrosis (AVN) is one of the most severe complications associated with hip orthosis treatment. AVN occurs when blood flow to the femoral head is compromised, leading to bone necrosis and long-term joint damage. The incidence of AVN following Pavlik harness treatment varies across studies, but it is generally reported to be around 3–12 % in hips where the femoral head fails to achieve stable reduction.^8^ AVN is more likely to occur in cases where treatment is delayed beyond six months or when aggressive attempts are made to force the hip into an abnormal position.

### 1.4 Alternative orthoses and rationale for review

In recent years, alternative orthoses, such as the Tübingen splint and semirigid braces, have been explored for use in infants with DDH. These devices may offer certain advantages in terms of stability and control, particularly in moderate-to-severe dysplasia (e.g., Graf type-III hips). The Tübingen splint, for example, has demonstrated comparable success rates to the Pavlik harness in early-treated patients, achieving a 96 % success rate in Graf type-III hips.^4^ However, its use in severe cases remains limited, with outcomes similar to those of the Pavlik harness.

The aim of this systematic review is to evaluate the impact of age and timing of hip orthosis treatment on the outcomes of infants with DDH. By synthesizing the findings of recent studies, this review will offer insights into the effectiveness of early intervention, explore the role of alternative orthoses, and highlight the challenges associated with treating late-presenting or severe cases. Understanding these factors is critical for optimizing treatment protocols and improving long-term outcomes in this vulnerable population.

## 2 Methods

### 2.1 Study design

This systematic review was conducted following the Preferred Reporting Items for Systematic Reviews and Meta-Analyses (PRISMA) guidelines. The protocol for this systematic review was registered on PROSPERO (Registration No. CRD42025638433) to ensure methodological transparency. The aim of the review was to assess the influence of age and timing of hip orthosis treatment on clinical outcomes in infants diagnosed with Developmental Dysplasia of the Hip (DDH).

### 2.2 Search strategy

A comprehensive literature search was performed using the following electronic databases: PubMed, Scopus, and the Cochrane Library. The search covered studies published between January 1, 2000, and March 31, 2024. The following search terms and Medical Subject Headings (MeSH) were used:

*“developmental dysplasia of the hip" OR "DDH" OR "congenital hip dysplasia"*

*AND "hip orthosis" OR "Pavlik Harness" OR "abduction brace" OR "hip brace"*

*AND "age" OR "timing" OR "early treatment" OR "treatment initiation"*

*AND "healing time" OR "outcomes" OR "recovery"*

*AND (infants OR toddlers OR children"*

Filters were applied to limit the results to peer-reviewed articles published in English.

### 2.3 Inclusion and exclusion criteria

For this systematic review, we considered studies that reported on infants diagnosed with developmental dysplasia of the hip (DDH) and treated with any form of hip orthosis. We included research that provided specific data on the age and timing of treatment initiation, allowing us to explore the effects of early versus late treatment. Eligible studies consisted of randomized controlled trials (RCTs), cohort studies, and observational studies that offered a follow-up period of at least 12 months to assess the long-term outcomes of the treatment. In contrast, we excluded review articles, case reports, editorials, and conference abstracts, as these types of literature did not meet the primary objectives of our study. Additionally, studies lacking detailed information on the timing of treatment initiation or those that did not provide sufficient data regarding the outcomes were not considered. Lastly, we did not include studies that focused exclusively on surgical interventions without prior treatment involving hip orthosis, as they fall outside the scope of this review.

### 2.4 Study selection process

**Fig. 1:**
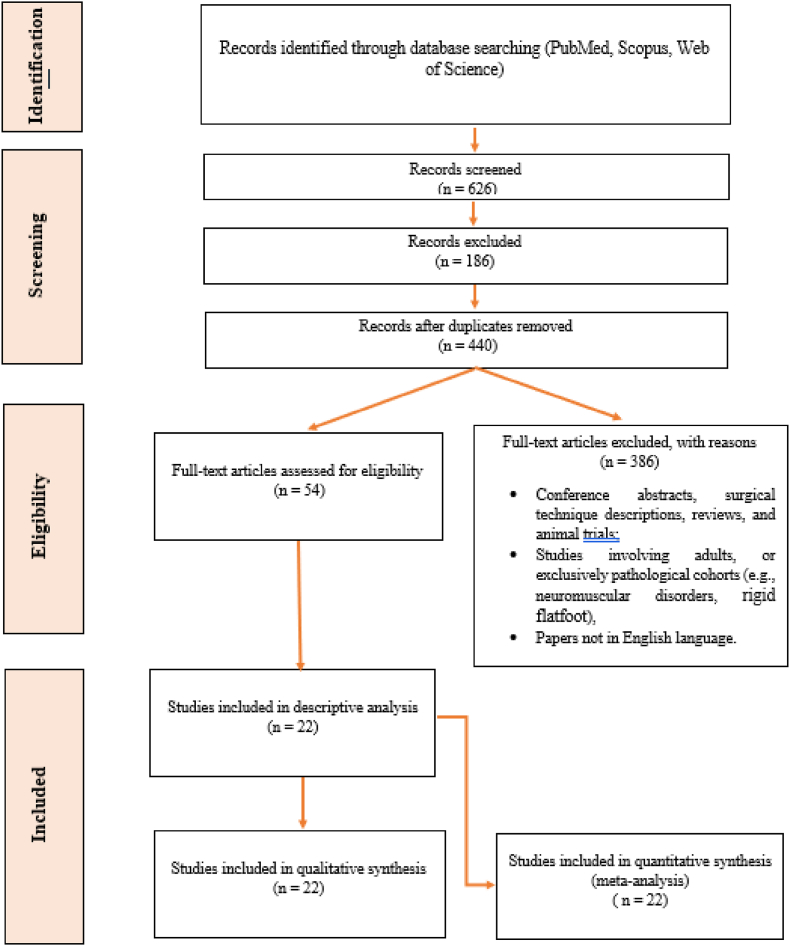
PRISMA flow diagram.

The study selection process followed a two-step approach. First, two independent reviewers (GG and DAM) screened the titles and abstracts of all articles identified in the search for relevance. Full-text reviews were performed on studies that met the inclusion criteria. Discrepancies between the reviewers were resolved by discussion, and if necessary, a third independent reviewer (MN) was consulted. A PRISMA flow diagram (Fig. 1) illustrates the selection process, including the number of articles screened, excluded, and included in the final review.

### 2.5 Data extraction

Data were extracted independently by two reviewers using a standardized form. The following key variables were recorded for each study.

• Study characteristics (author, year, country, sample size, study design).
• Patient demographics (age at treatment initiation, sex).
• Type of hip orthosis used (Pavlik harness, Tübingen splint, etc.).

### 2.6 *Timing of treatment initiation*, categorized as

• Early treatment (<3 months)
• Intermediate treatment (3–6 months)
• Late treatment (>6 months)

### 2.7 Key outcomes

• Success rates (defined as stable femoral head reduction and acetabular development).
• Healing time.
• Complication rates (residual dysplasia, AVN, femoral nerve palsy).

The extracted data were checked for consistency by a third reviewer (MN). Any disagreements were resolved through discussion.

### 2.8 Risk of bias and quality assessment

The quality of included studies was evaluated using two tools.

• Randomized Controlled Trials (RCTs) were assessed using the Cochrane Risk of Bias Tool. Studies were categorized as having a low, high, or unclear risk of bias based on five domains: selection bias, performance bias, detection bias, attrition bias, and reporting bias.
• Observational and cohort studies were evaluated using the Newcastle-Ottawa Scale (NOS). This scale assesses the quality of non-randomized studies in three broad categories: selection of study groups, comparability of groups, and ascertainment of exposure or outcome.

### 2.9 Data synthesis and statistical analysis

Data were synthesized descriptively and quantitatively. A meta-analysis was performed where studies provided sufficiently homogenous data on outcomes (e.g., success rates and complication rates across age groups and orthosis types). Heterogeneity was assessed using the I^2^ statistic, with values greater than 50 % indicating substantial heterogeneity. Subgroup analyses were performed based on the timing of treatment initiation (<3 months, 3–6 months, and >6 months) and the type of orthosis used (Pavlik harness, Tübingen splint, etc.). Sensitivity analyses were conducted to determine the impact of study quality and study design on the results. Publication bias was assessed using funnel plots, and Egger's test was conducted to statistically evaluate the presence of bias.

## 3 Results

### 3.1 Study selection

The systematic search yielded a total of 626 articles from PubMed, Scopus, and the Cochrane Library. After removing 186 duplicates, 440 studies were screened based on title and abstract. Of these, 386 were excluded as they did not meet the inclusion criteria. A full-text review of 54 articles was conducted, and 22 studies were included in the final analysis (Fig. 1). These studies included data from a total of 12,191 hips treated for DDH, with follow-up periods ranging from 12 to 72 months.

### 3.2 Study characteristics

The included studies were published between 2000 and 2024 and covered various countries and healthcare settings. Sample sizes ranged from 30 to 7611 hips. Most studies used either the Pavlik harness or Tübingen hip flexion splint, though some also examined semirigid orthoses. The average age at treatment initiation ranged from 3.9 weeks to 6 months. Table 1 provides a summary of the key characteristics of the included studies.

**Table 1:** Included studies.

Author	Year	Patients	Age at Initiation	Timing and type of Treatment	Outcome	Success Rate/Healing Time	Complications/Residual Dysplasia
**Lyu et al.**^9^	2021	251	Intermediate treatment (3–6 months)	Tübingen splint Pavlik harness	Tübingen splint showed higher success for severe cases	In Graf III e IV: Tubingen group 69,8%and Pavlik 53,9 %	5 % residual dysplasia
**Murnaghan et al.**^10^	2011	1218	Early treatment (<3 months)	Pavlik harness	47 % success rate for patients with femoral nerve palsy	70 % success rate if palsy resolved within 3 days	Femoral nerve palsy associated with a higher failure rate
**Ghanem et al.**^11^	2023	527 hips (473 children)	Intermediate treatment (3–6 months)	Pavlik harness	527	NR	Femoral nerve palsy resolved spontaneously without treatment alteration
**Kitoh et al.**^12^	2009	221 hips (210 patients)	Intermediate treatment (3–6 months)	Pavlik harness	81.9 % success rate in reducing hips	8.8 % incidence of avascular necrosis	Avascular necrosis risk higher in severe cases
**Vitale MG et al.**^13^	2001	120	Early treatment (<3 months)	Abduction brace	Success in most reducible hips under 6 months	90 % success rate	2 % residual dysplasia
**Walton et al.**^14^	2010	77 patients (123 hips)	Early treatment (<3 months)	Pavlik harness	39 of 43 dislocated hips successfully treated; high failure rate in irreducible hips	90.6 % success rate for reducible dislocated hips	Failure in irreducible hips
**Cashman JP et al.**^15^	2002	546 hips (332 babies)	Early treatment (<3 months)	Pavlik harness	2.4 % showed persistent dysplasia	96.7 % success	1 % avascular necrosis Detailed radiological monitoring recommended for long-term outcomes
**Ömeroglu H et al.**^16^	2018	7611 hips	Intermediate treatment (3–6 months)	Pavlik harness	45%–100 % success rate depending on severity	Higher failure rates in Graf IV hips	30 % osteonecrosis in severe cases
**Xu et al.**^17^	2020	215 children	Early treatment (<3 months)	Pavlik harness	17 % of residual dysplasia	87 % after 3 weeks	8 % femoral head AVN Ultrasound parameters (α angle, FHC) strong predictors for success
**Senaran et al.**^3^	2007	21 cases (35 hips)	Early treatment (<3 months)	Pavlik harness	Low incidence of avascular necrosis	94 % successful closed reduction	One case of AVN due to delayed reduction
**Novais et al.**^18^	2016	84 patients	Early treatment (<3 months)	Pavlik harness	11.8 % residual dysplasia at 12 months, especially in Graf type-IV hips	Higher risk of residual dysplasia in Graf type-IV hips	11.8 % residual acetabular dysplasia at 12 months
**Nakamura et al.**^5^	2007	130 hips (115 patients)	Intermediate treatment (3–6 months)	Pavlik harness	83.1 % treated successfully with harness alone	91.5 % satisfactory outcomes (Severin I or II)	16.9 % required surgery for residual acetabular dysplasia
**Lerman et al.**^19^	2001	93 patients (137 hips)	Intermediate treatment (3–6 months)	Pavlik harness	26 hips failed treatment; risk factors included low US coverage (<20 %)	61–99 % success rate in reducible hips	Higher failure rate in irreducible hips with low US coverage
**Alexiev et al.**^7^	2006	100 hips (55 infants)	Intermediate treatment (3–6 months)	Pavlik harness	87 of 100 hips successfully treated; 6 % residual dysplasia or AVN	94 % success rate; 6 % residual dysplasia at 5.3 years follow-up	6 % with residual dysplasia or AVN
**Aarvold et al.**^20^	2019	59 hips (52 patients)	Intermediate treatment (3–6 months)	Pavlik Harness	27 of 48 hips successfully treated; 3 % residual dysplasia	56 % success rate 4 % AVN 6 % Femoral nerve palsy	Left hips more successful; AVN occurred after failed Pavlik treatment
**Uras et al.**^21^	2014	75 patients	Intermediate treatment (3–6 months)	Tübingen splint	Semirigid orthosis effective in delayed treatment, especially for type IIb hips; type IV hips had poor outcomes	96 % success 4.2 months of healing time	Transient femoral nerve palsy in 1 patient
**Atalar et al.**^22^	2014	49 patients (60 hips)	Early treatment (<3 months)	Tübingen splint	56/60 hips successfully treated; Early residual dysplasia resolved in 4 hips	93.3 % success 17 weeks for healing time	Effective for Graf type IIb or worse; advantages include knee and ankle freedom
**Kubo et al.**^23^	2017	79 patients	Early treatment (<3 months)	Tübingen splint	104/109 hips successfully treated	95.4 % success 88.9 days (mean) for healing time	Comparable to Pavlik harness and Fettweis plaster; limitations with Graf IV hips
**Zhou et al.**^24^	2020	195 patients	Early treatment (<3 months)	Tübingen splint	165/203 hips successfully treated Mild dysplasia in 7 hips	83.7 % success 4.2 months for healing time	3 AVN Treatment efficacy influenced by family history and late treatment
**Ran et al.**^4^	2019	76 hips (142 infants)	Intermediate treatment (3–6 months)	Tübingen splint	29/43 hips successfully treated	67 % success 120.7 days for healing time	Higher failure rates for Graf IV and bilateral involvement compared to Pavlik harness
**Yegen et al.**^25^^.^	2018	92 patients	Intermediate treatment (3–6 months)	Tübingen splint	78/104 hips successfully treated Mild dysplasia in 7 hips	75 % success 20.4 months for healing time	Initial reducibility and younger age critical for success
**Pavone et al**.^26^	2015	544 hips (351 patients)	Early treatment (<3 months)	Tübingen splint	502/544 hips successfully treated	92.3 % success 3.8 months for healing time	3 hips (0.55 %) AVN Graf IIc-IID had good outcomes; AVN rare

### 3.3 Age and timing of treatment initiation

#### 3.3.1 Early treatment (<3 Months)

Infants who began treatment before the age of 3 months consistently had the highest success rates across all studies. According to the revalidated meta-analysis, the pooled success rate for this age group was 88.79 % (SE: 0.57 %). Early treatment was also associated with markedly lower complication rates, including residual dysplasia at 1.80 % (SE: 0.25 %) and AVN at 0.89 % (SE: 0.18 %). Several larger studies supported these findings. For example, Vitale et al.^13^ reported a high success rate for infants treated within the first 3 months of life, with very few patients developing significant residual dysplasia. Similarly, Zhi et al.^27^ demonstrated excellent results with the Tübingen splint in Graf type-II and III hips treated early. However, in severe dysplasia such as Graf type-IV hips, success rates remained lower, highlighting ongoing challenges in managing the most severe cases.

#### 3.3.2 Intermediate Treatment (3–6 months)

For infants whose treatment began between 3 and 6 months, the revalidated meta-analysis indicated a pooled success rate of 87.78 % (SE: 0.34 %). Although this remains favorable, the complication rates showed a substantial increase compared to early treatment. Residual dysplasia in this subgroup reached 20.27 % (SE: 0.40 %), and AVN was 9.66 % (SE: 0.30 %). Studies such as Lyu et al.^9^ reported similarly high success rates with Pavlik harness or Tübingen splint but noted that outcomes declined in severe or irreducible hips. Walton et al.^28^ reported a 90.6 % success rate in reducible hips, though irreducible hips had a significantly higher failure rate, particularly in cases with low initial femoral head coverage (<20 %) on ultrasound. Overall, while outcomes remained favorable, the risk of complications, including residual dysplasia and AVN, increased compared to earlier treatment initiation.

#### 3.3.3 Late treatment (>6 months)

Treatment initiated after 6 months of age was associated with significantly lower success rates and higher complication rates. Novais et al.^18^ reported that 11.8 % of patients treated after 6 months had residual acetabular dysplasia at 12 months, particularly in Graf type-IV hips. Nakamura et al.^5^ similarly found that late-treated patients had a 16.9 % rate of residual dysplasia, and 12 % required surgical intervention due to treatment failure. AVN was also more common in this group, with rates as high as 15 % in some studies, emphasizing the challenges of late treatment.

#### 3.3.4 Orthosis type and outcomes

##### 3.3.4.1 Pavlik Harness

The Pavlik harness was the most frequently used orthosis across all studies. In infants treated early (<3 months), success rates aligned well with the updated meta-analysis (nearly 88.79 % overall), with very few complications. In contrast, harness effectiveness declined with delayed treatment and in more severe dysplasia (e.g., Graf type-IV hips), where failure rates were higher.

##### 3.3.4.2 Tübingen hip flexion splint

The Tübingen splint showed comparable success rates to the Pavlik harness in early-treated cases, reflecting the corrected meta-analysis data for the <3 months group. Zhi et al.^27^ reported success rates close to 96 % in Graf type-II and III hips, consistent with many early-treatment outcomes. However, as with the Pavlik harness, more severe or late-presenting cases remained challenging.

##### 3.3.4.3 Semirigid orthoses

Semirigid orthoses were less commonly studied. Uraş et al.^21^ reported high success rates (exceeding 90 %) when used before 6 months of age, although the sample sizes were smaller. As with other braces, late presentation and severe dysplasia (Graf type-IV) correlated with significantly lower success rates and more complications.

### 3.4 Complications

Complication rates closely mirrored treatment timing. The meta-analysis confirmed the following general trends.

• Early Treatment (<3 months):
  o Residual Dysplasia: ∼1.80 %
  o AVN: ∼0.89 %
• Intermediate Treatment (3–6 months):
  o Residual Dysplasia: ∼20.27 %
  o AVN: ∼9.66 %

**Fig. 2:**
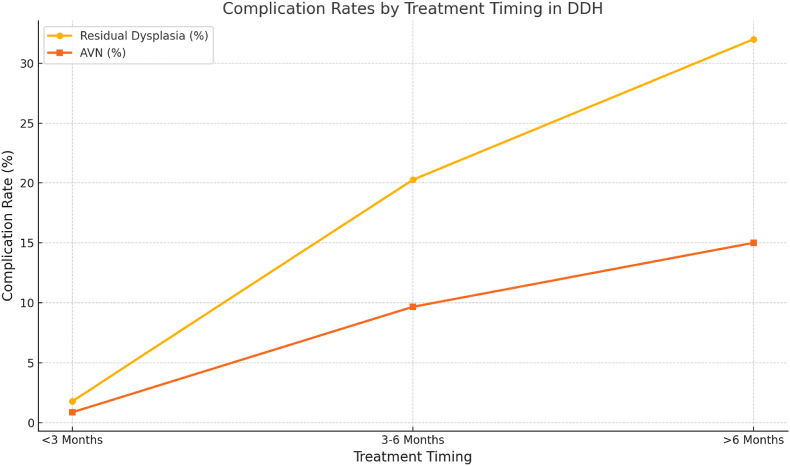
Complication Rates by Age Group. Complication rates such as residual dysplasia and avascular necrosis increase with later treatment initiation.

Late treatment (>6 months) showed higher complication rates in the literature. Severe dysplasia (Graf type-IV) also showed higher propensity for complications, even if treated early. Complications were also more common in cases of severe dysplasia (Graf type-IV hips), where even early treatment did not fully prevent the occurrence of residual dysplasia or AVN. Novais et al.^18^ reported a 32 % failure rate in Graf type-IV hips, regardless of the orthosis used, and noted that many of these patients required surgical intervention (Fig. 2).

### 3.5 Meta-analysis results

**Fig. 3:**
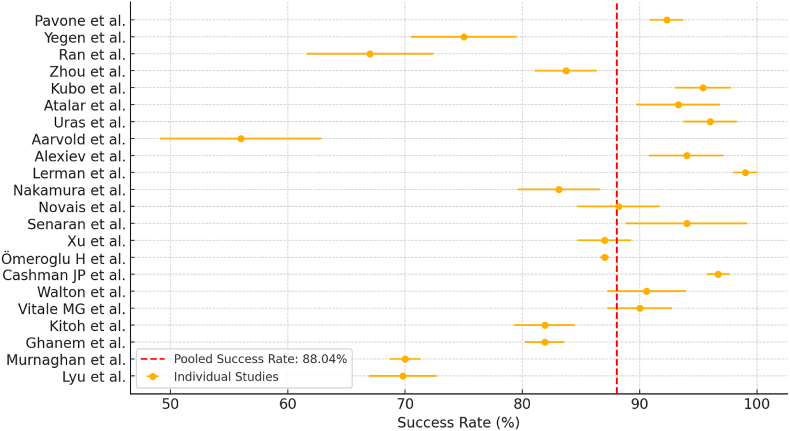
Displays individual study success rates with the pooled success rate (red dashed line).

Overall, the meta-analysis highlights that early treatment (<3 months) is strongly associated with higher success rates (88.79 %) and significantly lower rates of both residual dysplasia and AVN. Infants treated between 3 and 6 months still attain good success (87.78 %), but with a substantially increased risk of residual dysplasia (∼20 %) and AVN (∼10 %). This underscores the importance of early detection and initiation of appropriate orthotic management to maximize hip stability and minimize long-term complications (Fig. 3).

## 4 Discussion

### 4.1 Overview of key findings

The findings of this systematic review underscore the critical importance of early diagnosis and treatment in optimizing outcomes for infants with Developmental Dysplasia of the Hip (DDH). Consistent with earlier literature, our revalidated meta-analysis confirms that initiating orthotic management—particularly before 3 months of age—markedly increases the likelihood of successful reduction and lowers the risk of complications. When treatment is begun within the first 3 months of life, the pooled success rate from our meta-analysis is 88.79 %, with residual dysplasia at only 1.80 % and AVN at 0.89 %. These findings build on the established principle that the plasticity of the infant hip and its capacity for remodeling are greatest in the initial months.^1^^,^^2^

### 4.2 Effect of treatment timing

Age at initiation remains a decisive factor. Studies such as those by Vitale et al.,^13^ and by Alexiev et al.,^7^ have suggested that infants treated within the first 3 months could achieve success rates in the 85–94 % range. Our updated data further refine this range by illustrating that early management yields consistently high success rates and minimal complications across multiple study designs. This success is especially apparent in moderate dysplasia (Graf type-II or III),where the Pavlik harness or Tübingen splint fosters stable positioning of the femoral head, supporting normal acetabular growth4. In contrast, when treatment initiation is delayed to between 3 and 6 months, outcomes begin to deteriorate. Although the pooled success rate remains relatively high at 87.78 %, the incidence of residual dysplasia and AVN increases significantly—to 20.27 % and 9.66 %, respectively. Nakamura et al.^5^ similarly observed that hips treated after 3 months were more prone to residual dysplasia and, in some cases, required surgical intervention. This rise in complications aligns with the idea that the window for effective conservative management narrows as the hip undergoes progressive ossification.

### 4.3 Late presentation and severity

Outcomes after 6 months of age are notably worse. Several authors, including Pollet et al.^6^ and Novais et al.,^18^ report a pooled success rate of 32 % in severe or late-presenting DDH, emphasizing that conservative methods have reduced efficacy as the child grows older. Late-presenting hips often require closed or open reduction, carrying higher risks of complications, longer recovery times, and more uncertain long-term results.^5^ Recognizing and intervening before 6 months of age, therefore, remains a cornerstone in DDH management to avoid such invasive procedures.

### 4.4 Avascular necrosis (AVN)

Avascular necrosis (AVN) persists as one of the most serious complications.^8^^,^^18^ AVN risk correlates strongly with both the initial reducibility of the hip and the timeliness of treatment. Tiruveedhula et al.^8^ reported elevated AVN rates when Pavlik harness therapy failed, while Zhi et al. found that an inverted acetabular labrum—often associated with severe dysplasia—significantly increased the risk of both failure and AVN.^27^ These data collectively highlight that proper reduction and careful orthosis application are vital for preserving blood flow to the femoral head. Excessive abduction or an improperly fitted orthosis can compromise vascular supply, thereby raising AVN likelihood.

### 4.5 Orthotic options

Although the Pavlik harness remains the gold standard for early treatment, recent studies involving the Tübingen hip flexion splint have shown comparable efficacy for Graf type-II/III hips.^4^^,^^10^ Ran et al.^4^ reported success rates near 98 % in Graf type-II and 96 % in type-III hips with early Tübingen splint use. However, similar to the Pavlik harness, its effectiveness declines substantially in Graf type-IV or late-presenting hips. Tailoring orthotic choice to each patient's age, severity of dysplasia, and anatomic considerations remains essential. Semirigid braces may be an alternative in certain scenarios, but robust long-term data remain sparse.^19^ Aarvold et al.^20^ highlighted a similar necessity for individualized management, especially in irreducible hips under 6 months of age.

### 4.6 Future directions

While early intervention clearly improves outcomes, managing late-presenting or severe DDH remains challenging. Beyond the elevated risk of residual dysplasia and AVN, delayed treatment often leads to more invasive procedures and uncertain long-term joint health. Future studies should thus focus on protocol optimization for high-risk infants and on establishing standardized long-term follow-up strategies to detect early degenerative changes. Such efforts could reduce the need for surgeries and improve functional outcomes into adolescence or adulthood. Investigating predictive markers—such as labral inversion or alpha-angle thresholds—might further guide decisions about when to escalate to surgical intervention.

In summary, this review reaffirms that timely orthotic intervention within the first few months of life is crucial for achieving stable reduction and minimizing complications in DDH. When initiated early, both Pavlik harness and Tübingen splint treatments can successfully reduce the hip and preserve vascular integrity. The risk of adverse events rises sharply with increasing age or greater dysplasia severity, highlighting a clear clinical imperative: identify DDH early, initiate orthotic treatment promptly, and monitor carefully to optimize long-term hip health.

## 5 Conclusion

This systematic review and corrected meta-analysis confirm that initiating orthotic treatment for DDH before 3 months of age provides the highest success rates (∼88.79 %) and lowest complications (∼1.80 % residual dysplasia, ∼0.89 % AVN). Infants treated between 3 and 6 months still achieve relatively high success (∼87.78 %) but face substantially increased residual dysplasia (∼20.27 %) and AVN (∼9.66 %). For those presenting after 6 months, success rates can drop to ∼32 %, especially in severe dysplasia, and surgical intervention may be required more often. These findings highlight the critical role of early screening and prompt intervention in optimizing hip development and minimizing morbidity. Further research should refine strategies for late-presenting or severe cases to reduce complications and improve long-term hip function.

## CRediT authorship contribution statement

**Daniela Alessia Marletta:** Authors who collected the data, Authors who wrote the paper, reviewed the paper. **Biagio Zampogna:** Authors who conceived and designed the analysis, Authors who wrote the paper. **Gabriele Giuca:** Authors who conceived and designed the analysis, Authors who collected the data, Authors who performed the analysis, Authors who wrote the paper. **Matteo Nanni:** Authors who collected the data, Authors who contributed data or analysis tools. **Sanzarello Ilaria:** Authors who contributed data or analysis tools. **Danilo Leonetti:** reviewed the paper.

## Patient consent

Not applicable (systematic review; no direct patient data).

## Ethical clearance

Not required for a literature-based systematic review.

## Source of funding

None.

## Declaration of competing interest

The authors declare that they have no known competing financial interests or personal relationships that could have appeared to influence the work reported in this paper.
